# The necessity and optimal time for performing pars plana vitrectomy in acute retinal necrosis patients

**DOI:** 10.1186/s12886-018-0674-9

**Published:** 2018-01-22

**Authors:** Shulin Liu, Desai Wang, Xuedong Zhang

**Affiliations:** Ophthalmology Department, Chongqing Key Laboratory of Ophthalmology and Chongqing Eye Institute, The First Affiliated Hospital of Chongqing Medical University, Chongqing, People’s Republic of China

**Keywords:** Acute retinal necrosis, Pars plana vitrectomy, Retinal detachment

## Abstract

**Background:**

To compare the efficacy of pars plana vitrectomy (PPV) at different time points to treat acute retinal necrosis (ARN) and to investigate the necessity of PPV for ARN.

**Methods:**

A retrospective review of the treatment options and outcomes of the ARN patients was performed. Thirty ARN patients (34 eyes) were included in this study. The eyes were divided into 3 groups depending on the treatment administered. In the medically treated group, there was no retinal detachment (RD) at the first visit. The routine group patients were treated with systemic antiviral medications, as well as with intravitreal antiviral injections. In the early PPV treatment group, there was no RD at the first visit. The early PPV treatment group patients were treated with systemic antiviral medications and PPV plus silicone oil tamponade and intravitreal injection. In the PPV group, there was RD at the first visit. The PPV group patients were treated with systemic antiviral medications and PPV plus silicone oil tamponade and intravitreal injection.

**Results:**

In the medically treated group, the mean baseline best corrected visual acuity (BCVA) (logMAR) was 1.38 ± 0.35. The BCVA was 1.21 ± 0.36 at the last visit for the medically treated group. In this group, one eye (12.5%) developed RD after 1 month of treatment. In the early PPV treatment group, the mean BCVA (logMAR) was 1.68 ± 0.26. The BCVA was 1.83 ± 0.21 at the last visit for the early PPV group. In this group, five eyes (29.4%) had recurrent RD before silicone oil removal. In the PPV group, the mean BCVA (logMAR) was 2.0 ± 0.35. The BCVA was 1.72 ± 0.34 at the last visit for the PPV group. In this group, one eye (11.1%) had recurrent RD before silicone oil removal. There were no significant differences among the three groups in the baseline BCVA and the BCVA at the last visit (p>0.05). There were no significant differences between the early PPV group and the PPV group in the recurrent RD rates (*p* = 0.38).

**Conclusions:**

Prophylactic PPV showed no difference in recurrent RD rates or better BCVA. Therefore, prophylactic vitrectomy cannot prevent RD nor improve the prognosis of ARN based on our research.

## Background

Acute retinal necrosis (ARN) was first reported in Japan in 1971 by Urayama [1]. Acute retinal necrosis is a rare disease, manifesting as panuveitis and retinal vasculitis, and then progressing to diffuse retinal necrosis. Acute retinal necrosis will eventually result in rhegmatogenous retinal detachment (RD). The pathogenic cause of ARN is a human herpes virus, such as the varicella zoster virus (VZV) or the herpes simplex virus (HSV) [2–4]. ARN affects both immunocompromised and immunocompetent people regardless of gender or age.

The most recognized treatment regimen of ARN is intravenous acyclovir at 10 mg/kg every 8 h for 7 to 10 days, followed by an oral antiviral medication [3, 5–7]. An immediate therapeutic dose in the vitreous body can be achieved by intravitreal antiviral injection, and this therapy has been increasingly adopted. Other adjunctive treatment options, such as laser to prevent RD or systemic corticosteroids, have also been described to treat vitritis, optic nerve head neuritis and vasculitis [3, 7].

Despite prompt treatment, the prognosis and visual outcomes of ARN are still poor. Retinal detachment is one of the main causes of this poor result [2, 8]. Pars plana vitrectomy (PPV) is generally carried out after RD; however, some researchers have suggested early PPV treatment may help prevent RD [3, 8]. Nevertheless, prophylactic PPV is still controversial and is the research subject of our study.

## Methods

The records of patients who were diagnosed with ARN between April 2010 and February 2017 were analyzed retrospectively. These cases were evaluated at the ophthalmology department of the First Affiliated Hospital of Chongqing Medical University. Our study was approved by the local institutional review board of the First Affiliated Hospital of Chongqing Medical University, and followed the statements of the Declaration of Helsinki.

ARN was diagnosed according to the American Uveitis Society Diagnostic Criteria (1994) [9]. All the patients also got a TORCH (Toxoplasmosis, Rubella, Cytomegalovirus, HSV) test for vitreous and serum. Then the quotients of the relative amount of antibodies against herpes antigens in the vitreous humor and serum (Goldmann–Witmer coefficient) was calculated: (antibody titer against viral antigens in the vitreous/IgG amount in the vitreous humor)/(antibody titer against viral antigens in the serum/ IgG amount in the serum). If the Goldmann-Witmer coefficient was larger than 6 for cytomegalovirus (CMV), the patient was diagnosed as CMV infection and exclude from the study [10]. Based on the patient’s clinical presentation at the first visit and their treatment options, the eyes were divided into one of three groups: a medically treated group, an early PPV treatment group, and a routine PPV treatment group. In the medically treated group, the eyes had no RD noted at the first visit and PPV was not initially performed. In the early PPV treatment group, the eyes had no RD noted at the first visit, and these patients received prophylactic PPV surgery. In the routine PPV treatment, the eyes had RD noted at the first visit and the patients received PPV surgery. All the patients in the study received intravenous acyclovir (10 mg/kg, administered every 8 h) treatment immediately after the diagnosis of ARN. After 10 days of intravenous antiviral treatment, the patients were then switched to oral acyclovir, which they received for 6 months. The patients also received systemic steroids and topical ophthalmic steroids, as well as topical ophthalmic cycloplegic. Systemic steroids were started 24–48 h later after the initiation of antiviral treatment. Ocular examinations included best corrected visual acuity (BCVA), slit-lamp examination, dilated fundus examination, and intraocular pressure. Auxiliary examinations such as B-scan ultrasound and optical coherence tomography were also performed.

PPV was performed by a skilled surgeon using a 23-gauge standard three-port system. Silicone oil was injected and endolaser photocoagulation was applied at the junction of the normal and affected retina. All the patients received 3 mg intravitreal injection of ganciclovir just before completion of the surgery.

### Statistical analysis

Statistical analysis was performed with SPSS version 20.0 (SPSS Inc., Chicago, Illinois, USA). For statistical analysis, the BCVA was converted to a logarithm of the minimum angle of resolution (logMAR). The counting finger was set as 2.6 (logMAR), hand motion was set as 2.9 (logMAR), light perception was set as 3.1 (logMAR), and no light perception was set as 3.4 (logMAR) [11]. Differences in visual acuity among groups were analyzed with independent samples *t*-test. Differences in the RD ratio among groups were analyzed using the chi-square test. The level of significance adopted in our study was less than 0.05.

## Results

A total of 30 patients (34 eyes) were included in the study. Of the 30 patients, 25 (83.3%) were male and 5 (16.7%) were female. Their age was 49.3 ± 11.0 years. Four patients (13.3%) had bilateral involvement. The time between disease onset and the first visit to our department was 27.1 ± 14.3 days. The follow-up period ranged from 2 to 30 months. Eight eyes were included in the medically treated group, 17 eyes were included in the early PPV treatment group, and 9 eyes were included in the medically treated group.

In the medically treated group, the BCVA before treatment was 1.38 ± 0.35 (logMAR). The patients received both systemic and intravitreal antiviral injection. All the patients received 1–3 injections, with an average 1.5 injections. Necrosis was confined and inflammation improved in 7 eyes. Only 1 eye (12.5%) developed RD, which occurred 1 month after the initiation of treatment. This eye then underwent PPV and silicone oil tamponade. At the last visit, the BCVA was 1.21 ± 0.36. Seven eyes (87.5%) had equal or better BCVA than that before treatment.

**Fig. 1:**
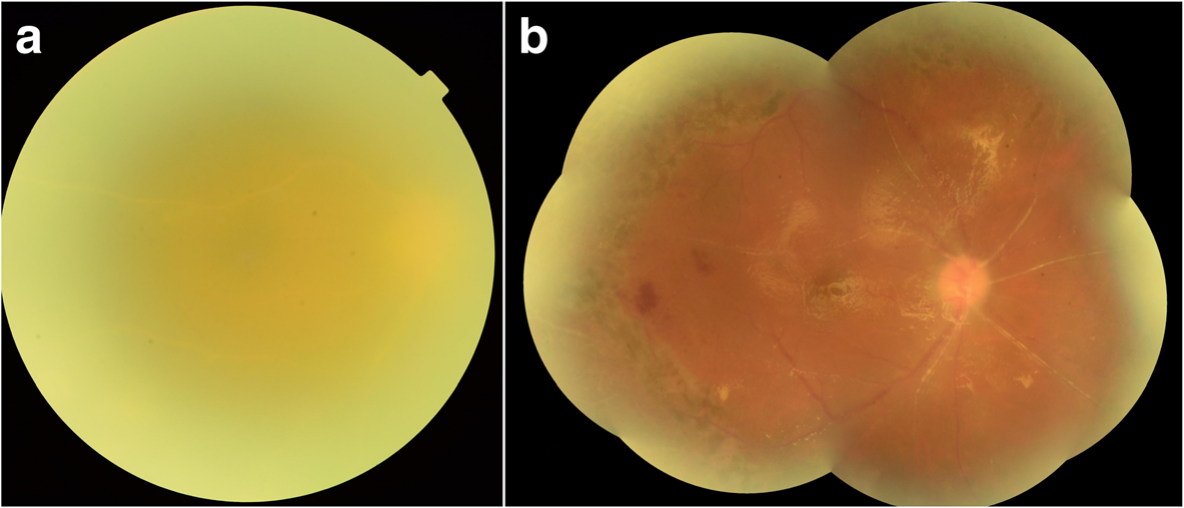
Fundus photograph demonstrated the changes of the retina in a patient in early PPV group. **a** Serous vitritis obscured the fundus before PPV. **b** The retina after PPV + silicone oil tamponade + endolaser photocoagulation. There were many peripheral retinal scars due to photocoagulation. Arterial occlusion could be seen. The BCVA improved from counting finger to 0.92 (logMAR) after PPV

In early PPV treatment group, BCVA before treatment was 1.68 ± 0.26 (logMAR). At the last visit, the BCVA was 1.83 ± 0.21 and sixteen eyes (94.1%) had attached retinas. Eight eyes (47.1%) had equal or better BCVA than that before treatment (Fig. 1). Five eyes (29.4%) had recurrent RD even before removal of silicone oil. Among them, 2 eyes had reattached retina after scleral buckling surgery. One eye had a reattached retina after silicone oil replacement surgery. One eye had a reattached retina after scleral buckling surgery; however, the RD reoccurred after silicone oil removal surgery, thus silicone oil tamponade surgery was carried out for a second time. One patient refused any further surgery to treat the RD. Only one eye underwent silicone oil removal without recurrent RD.

In the routine PPV treatment group, BCVA before treatment was 2.0 ± 0.35 (logMAR). At the last visit, the BCVA was 1.72 ± 0.34 and all the eyes had attached retinas. Six eyes (66.7%) had equal or better BCVA than that before treatment. One eye (11.1%) had recurrent RD before the removal of silicone oil, and its retina reattached after silicone oil replacement surgery. Two eyes in the group had already undergone silicone oil removal.

During the follow-up period, proliferative vitreoretinopathy (PVR) developed in almost all the eyes. Five eyes (29.4%) in the early PPV group and 1 eye (11.1%) in the medically treated group had recurrent RD because of PVR, and these eyes required further surgeries. Surgical corrections for PVR include membrane peeling, silicone oil replacement and further endolaser photocoagulation. Other complications such as cataracts, optic atrophy, and ocular atrophy also developed in the patients. Severe cataracts developed and needed phacoemulsification in 3 eyes in the early PPV group and 3 eyes in the routine PPV group after PPV. Only 1 eye in the early PPV group had posterior chamber intraocular lens implanted. Optic atrophy developed in 3 eyes in the early PPV group, 2 eyes in the routine PPV group, and 1 eye in the medically treated group. The patient who declined surgery after recurrent RD developed ocular atrophy.

Silicone oil removal was only performed in cases with retinal reattachment and without severe PVR. Before surgery, additional photocoagulation was applied around the area where the retinal breaks and membranes were present. Only one eye (5.9%) in the early PPV group and 2 eyes (22.2%) in the routine PPV group underwent silicone oil removal, and the retinas remained reattached.

The baseline and final BCVA for the three groups are listed in Table 1. There was no statistically significant difference in baseline or final BCVA among these groups. Moreover, there was no statistically significant difference between the rate of recurrent RD in the early PPV and the routine PPV group.

**Table 1:** The baseline and final BCVA of the three groups of patients

	BCVA				
Patients number	NLP	LP-1.0	0.5–1.0(include 1.0)	0.3–0.5(include 0.5)	≤0.3
Group 1(before treatment)	0	4	3	1	0
Group 1(after treatment)	0	1	2	3	2
Group 2(before treatment)	0	8	7	2	0
Group 2(after treatment)	0	13	3	0	1
Group 3(before treatment)	0	6	3	0	0
Group 3(after treatment)	0	5	3	0	1

The BCVA was documented as LogMAR in the table. *NLP* no light perception, *LP* light perception

## Discussion

ARN is a rare disease, thus large randomized clinical trials (RCT) are difficult to perform. Currently, the available treatment options are based on the results from a small number of retrospective studies or case reports. However, even with proper treatment, the prognosis for ARN is very poor. One study reported the BCVA to be worse than 20/200 after 6 months of the disease in nearly 50% of the patients [12]. RD is the most common cause of the poor prognosis. Other causes include optic atrophy, vitreous opacity, epiretinal membrane, retinal ischemia and macular edema [6, 13]. If left untreated, nearly 70% of the patients will develop ARN in the other eye [14], and ARN will occur within a few months [7, 14].

As the prognosis for ARN is poor, early diagnosis is critical. The diagnostic criteria for ARN published in 1994 mainly focused on clinical presentations [15]. More than 20 years later, new treatment techniques, such as PCR, have been developed. Many ophthalmologists now will use PCR for the diagnosis and determination of the pathogen involved in ARN. However, antiviral treatment should never be delayed while waiting for the PCR results.

In addition to systemic antiviral treatment, comparative studies have found that the combination of intravitreal therapy can reduce the incidence of RD and improve vision [16–18]. These previous studies used the drug foscarnet, at a 2.4 mg dose. In our clinic, we use ganciclovir, 3 mg, for intravitreal injection. However, intravitreal injection should never be used alone, as it cannot prevent the development of ANR in the contralateral eye and does not treat a possible concomitant herpetic encephalitis or meningits. In our study, just before completion of the PPV surgery, we performed intravitreal injection in the early PPV and the routine PPV group. In the medically treated group, intravitreal injection was performed on the first day of admission. If the inflammation was not fully controlled, further injections were performed 3 days apart.

The incidence of RD in ARN patients is high. Necrosis lesions first start peripherally and may have a circumferential spread, finally influencing the posterior pole of the retina, even with adequate diagnosis and antiviral treatment [19]. Therefore, RD can occur even after the resolution of ARN [20]. Some ophthalmologists recommended early PPV before RD to treat ARN. Early PPV can remove the inflammatory factors, relieve vitreous traction, and create a chance for complete laser photocoagulation after the vitreous opacities are cleared. Until now, 4 studies [2, 21–23] have researched the visual and anatomic outcomes after early PPV. However, their rating of the level of evidence were not high according to the rating scale based on the British Centre for Evidence-Based Medicine [24]. A level II rating was assigned to well-designed case control and cohort studies, and poor-quality randomized studies; and a level III rating was assigned to case series, case reports, and poor-quality cohort and case-control studies. Among the 4 studies, only 1 study was level II [21]. The level II study recruited 104 eyes with ARN and found no anatomic or visual benefit for early PPV. The other 3 level III studies found a possible benefit in reducing the frequency of RD, but no visual benefit. Our study also suggested that early PPV may not provide better outcomes compared with the routine PPV or the nonsurgical groups. In the early PPV group, surgery was performed before RD, and the vitreous was completely removed. However, we found that PVR developed in nearly every patient, even in the early PPV group. Severe PVR resulted in recurrent RD and was the main reason for the failure of the surgeries. These results further confirmed the destructive nature and very poor prognosis of this disease. The prevention of PVR in ARN patients warrants further research and may be the target of treatment in the future.

Before the end of the early PPV surgery, laser photocoagulation was performed. Prophylactic laser in the managing of ARN remains controversial. One report suggested that laser photocoagulation could not prevent RD [25]. However, the patients in that study did not have PPV surgery. The vitritis may prevent the complete of laser photocoagulation. Thus, the effectiveness of laser photocoagulation requires further research.

There are many complications of ARN, such as PVR, cataracts, and optic atrophy. Occlusive vasculitis in ARN patients will lead to retinal ischemia [19]. The affected retina becomes thinned and atrophic permanently [26]. Thus, even without RD, the visual prognosis is not good.

The present study has some limitations. First, it has a small sample size due to the rarity of ARN. Second, selection bias may be present. The patients with mild vitritis tend to be treated with systemic and intravitreal antiviral drugs first. Those with severe vitritis tend to be treated with early PPV. Nevertheless, there was no significant difference among these groups in the baseline BCVA. Furthermore, follow-up time varied among the patients. The longer the follow-up time, the more complications would develop, especially PVR. As it is impossible to conduct a multicenter RCT regarding ARN, this retrospective study can provide new evidence in the management of ARN.

## Conclusion

In conclusion, this study suggested that early PPV may not provide better outcomes compared with the routine PPV or the medically treated groups. Both PVR development rate and recurrent RD rate were high in ARN patients with different treatment protocols. Clinically, despite our efforts, their prognosis was poor. Further studies are needed to investigate the therapeutic method to decrease the development of PVR.

## Abbreviations

ARN: Acute retinal necrosis
BCVA: Best corrected visual acuity
RD: Retinal detachment
